# Contraception and Unintended Pregnancy among Unmarried Female University Students: A Cross-sectional Study from China

**DOI:** 10.1371/journal.pone.0130212

**Published:** 2015-06-19

**Authors:** Hongjing Wang, Lu Long, Hui Cai, Yue Wu, Jing Xu, Chang Shu, Peng Wang, Bo Li, Qinyu Wei, Xuejun Shang, Xueyi Wang, Meimei Zhang, Chengliang Xiong, Ping Yin

**Affiliations:** 1 Department of medical affairs, Gansu Provincial Hospital, Lanzhou, Gansu, Chin; 2 Department of Epidemiology and Biostatistics, School of Public Health, Tongji Medical College, Huazhong University of Science and Technology, Wuhan, Hubei, China; 3 Gansu Provincial Hospital, Lanzhou, Gansu, China; 4 The Public Management Teaching and Research Section, Humanities School, Henan Universities of Traditional Chinese Medicine, Zhengzhou, Henan, China; 5 Institute of Laboratory Medicine, Jinling Hospital, Nanjing University School of Medicine, Nanjing, Jiangsu, China; 6 Institute for Population and Family Planning of Chongqing City, Sexual Health Education Research Center, Chongqing, China; 7 Capital Normal University, Sexual Health Education Research Center, Beijing, China; 8 Institute of Family Planning, Tongji Medical College, Huazhong University of Science and Technology, Wuhan, Hubei, China; Karolinska Institutet, SWEDEN

## Abstract

This study aims to understand the level of contraceptive knowledge and attitudes towards contraception, and then to explore the association between the contraceptive behavior and unintended pregnancy in unmarried female university students in China. A cross-sectional study was conducted of university students in 49 universities across 7 cities in China from September 2007 to January 2008. We distributed 74,800 questionnaires, of which 69,842 were returned. In this paper, the data from 35,383 unmarried female university students were analyzed. The prevalence of sexual intercourse in unmarried female university students was 10.2%. The prevalence of unintended pregnancy in those sexually active female university students, was 31.8%. Among students with pregnancy, 53.5% experienced two or more pregnancies. 28.3% of the students with sexual intercourse reported that they always adopted contraceptive methods, and of those 82.9% chose to use male condoms. The majority (83.9%) of students with unintended pregnancy chose to terminate the latest pregnancy by surgical abortion or medical abortion. The contraceptive knowledge level of students who experienced unintended pregnancy was lower than those who did not. In China, about one third of unmarried female students with sexual intercourse experience unintended pregnancy. A variety of contraceptive methods are adopted, but the frequency of contraceptive use is low. Most of unmarried female students who experienced unintended pregnancy would choose to terminate the pregnancy with surgical or medical abortion. University students, especially the ones who have experienced unintended pregnancy, lack contraceptive and reproductive health knowledge.

## Introduction

With the development of the economy and improving access to the internet and social media, people in China are becoming more open-minded and the values they hold are becoming more diversified [1]. The attitudes of Chinese youth to marriage, family and sex have changed substantially in recent years [1]. Their attitudes towards the sex have become more and more indiscreet and premarital sexual intercourse have become more common[2]. In 2004 Shah et al showed that two fifths of young people aged 15 to 24 years in rural areas of Sichuan, China, had engaged in premarital sex[3]. A high incidence (15%) of premarital sex among university students has also been reported[4].Today, there are 30 million university students in China[5]. After experiencing the pressure of college entrance examinations, these students have entered the next stage of lives. They become more relaxed and more active in their personal lives as well as more open to the idea of premarital sexual intercourse. These factors lead to premarital sexual intercourse becoming common among university students [6–8]. In 2005, the Chinese Ministry of Education issued the new “The Regulation of Student Management in Regular University”, and lifted the ban on the marriage of university students. This, to a certain degree, might also contribute to the increasingly common premarital sexual intercourse and off campus cohabitation among university students [7,9,10].

Today’s university students will be the workforce which leads tomorrow’s social development in China. Their health as students will have an impact on their health tomorrow being the workforce of the society, and hence have an impact on the development of China in the future. Safe sex and prevention of unintended pregnancy are necessary measures to maintain good health among university students. Knowledge of reproduction and contraception are needed to ensure these are achieved. University students’ knowledge of reproduction and contraception needs to be evaluated, so that relevant health education and consulting can be provided. Zhang et al’s small sample study on sexual attitudes and activities in China concluded that Chinese university students had inadequate understanding of the harms that unsafe sexual activity, unintended pregnancy may cause to their health [2]. To date, there has not been a study with a sufficient sample size and a nationwide scope to explore the knowledge of reproductive health and contraception in Chinese university students. In the present work, a cross sectional study was conducted in the 49 universities in 7 cities from the different regions of China to address this issue.

Kelly’s study showed that women had a disadvantage and unequal status as compared to men when negotiating sexual encounters and adopting contraceptive methods[11]. Some unmarried female students worried that if they didn’t engaged in sexual intercourse, which would affect their relationships; also they couldn’t have a good communication with their partners, perhaps resulting unsafe sex intercourse. Unsafe sex in turn increases incidence of unintended pregnancy and abortion, as well as the risk of sexually transmitted infections (STIs) [12–14]. We believe that female students need a good understanding of contraception, sexual intercourse and unintended pregnancy. This paper is focused on the contraceptive knowledge, contraception behavior and unintended pregnancy in unmarried female university students in China.

## Methods

### Ethical Consideration

This work was supported by The Chinese “11th Five-Year Plan” Supporting Science and Technology Project [No. 2006BAI15B00]. The study was approved by the ethical committee of School of Public Health, Tongji Medical College, Huazhong University of Science and Technology, Wuhan, China (No. [2006]04) (S1 File). Participation in this study was voluntary and confidentiality of the information was assured. All participants provided written informed consent. We also obtained consent from the departments of student affairs of the relevant universities.

### Participants

The survey was conducted from September 2007 to January 2008. The stratified cluster sampling method was used to enroll participants according to the type of universities and the levels of education to ensure that the sample was nationally representative. Among 49 universities, 12 were in North China (6 in Beijing and 6 in Changchun), 16 in West China (8 in Chongqing and 8 Chengdu), 7 in East China (7 in Nanjing), 7 in South China (7 in Nanning), and 7 in Central China (7 in Wuhan). In each university, 200, 150 and 50 students per grade were selected from undergraduate, Master’s degree program and doctoral program respectively. All the selected students completed the registration and provided informed consent. We distributed 78,400 questionnaires and collected 69,842 valid questionnaires. The validation of the questionnaire was decided according to the overall answers to key questions. The response rate was 89.1%. After exclusion of 2520 married students, 67,322 unmarried students, 31,939 males and 35,383 females, were included in the investigation. The questionnaires of 35,383 female students were analyzed in this paper.

### Measurement

The questionnaire for the investigation was developed by referring to the questionnaires of published research on reproduction health[15,16]. The content of the questionnaire was defined by the repeated discussion with experts in the concerned fields. A pilot-test was conducted in the 800 students in 2 universities in Wuhan before the final survey, and the questionnaire was evaluated for the reliability (Cronbach’s alpha coefficient was 0.91) and validity (Goodness of Fit Index was 0.87). After the further modifications and revisions, the content of the final questionnaire included the basic characteristics of the students, the knowledge of contraception and the attitude towards contraception, the contraceptive methods used, as well as unintended pregnancy (S2 File).

The knowledge and attitude section included “the primary consideration for choosing contraceptive methods”, “the perceived side effects of oral contraception pills (OCPs)”,“whether emergency contraception (EC) can be a substitute for regular contraception”, “the methods of emergency contraception”, “whether abortion influences women's physical and mental health”. The contraceptive methods used section included “the frequency of contraceptive methods use”, “the contraceptive methods”. The other items included “the main reason for not using contraception”, “how to deal with unintended pregnancy” and so on. Before the field investigation, the investigators were trained uniformly. Because the topic is highly sensitive, the questionnaire was anonymous and self-administered. The questionnaires were delivered to the selected class as a unit through the universities. The systematic data check was done before statistical analysis to ensure that the facts are complete and reliable.

### Analysis

The descriptive analysis was applied to describe the basic characteristics, as well as the prevalence of premarital sexual intercourse and that of unintended pregnancy of the students. A logistic regression was carried out to determine the association between unintended pregnancy and basic characteristics, and odds ratios (OR) and 95% confidence intervals (CI) were calculated. Chi-square test and Fisher’s exact test were used to compare the difference in the knowledge of contraception and attitude towards contraception, as well as the types of contraceptive methods used and frequency of contraceptive methods use between the female students with unintended pregnancy and those without. For the students who didn’t adopt contraceptive methods, the reasons for not using were analyzed with Chi-square test and Fisher’s exact test too. Data analysis was carried out using SAS 9.1.3 (SAS Institute Inc., Cary, NC), *p*-value less than 0.05 was considered statistically significant.

## Results

### Basic characteristics at the first sexual intercouse

The total number of unmarried female students was 35,383. Among them, 29,814(84.3%) were undergraduates, 4,950(14.0%) were master degree graduate students, and 619(1.7%) were doctoral degree graduate students. 10,739(30.4%) students were in medical universities or medical colleges. The mean age was 21.0 with SD of 6.2.

### Sexual intercourse and unintended pregnancy

According to our survey, 3,595(10.2%) of 35,383 unmarried female students engaged in sexual intercourse, and 1,142(31.8%) of these experienced unintended pregnancy. Among those who experienced unintended pregnancy, 531(59.5%) experienced one, 236(26.4%) experienced two and 126(14.1%) experienced three or more. There were 249 missing values for the number of unintended pregnancies.

According to the logistic regression analysis, the occurrence of unintended pregnancy was associated with the students’ characteristics at the first sexual intercourse (Table 1). Those who were younger than 20 or aged from 20 to 22 had higher risk of unintended pregnancies (OR = 1.5, 95% CI: 1.2~2.1; OR = 1.3, 95% CI: 1.1~1.6, respectively) compared to students who were older than 22. Undergraduate students had a higher occurrence of unintended pregnancy than the doctoral degree graduate students (OR = 1.2, 95% CI: 1.1~1.7). Non-medical students had a higher risk of having unintended pregnancy than medical students (OR = 1.2, 95% CI: 1.1~1.4).

**Table 1:** The association between unintended pregnancy and characteristics at the first sexual intercourse.

Basic characteristics at the first sexual intercourse	OR	95%CI
Age		
<20	1.5^Δ^	1.2~2.1
20~	1.3Δ	1.1~1.6
22~30	1.0	1.0
Education		
Undergraduate	1.2Δ	1.1~1.6
Master	0.7	0.5~1.1
Doctor	1.0	1.0
Discipline		
Non-medicine	1.2Δ	1.1,1.4)
Medicine	1.0	1.0

^Δ:^ *P*<0.05.

### Contraceptive knowledge and attitude

As shown in Table 2, in women who had sexual intercourse, there was a significant difference in the primary consideration for choosing contraceptive methods between the students with experience of unintended pregnancy and those without (*p*<0.05). The percentage of students who primarily considered the effectiveness of contraceptive methods was only 33.8%. In 45.6% of students, the primary consideration was the safety of methods. 20.6% of the students considered the feeling of sex and the convenience of buying and/or using contraceptive tools first. Concerning the side effects of OCPs, 68.5% of the students believed that OCPs could affect the regularity of the menstrual cycle, 62.2% believed OCPs could affect fertility, 39.6% believed OCPs could cause nausea or vomiting, and 38.2% believed OCPs might cause weight gain. 75.3% of the students understand EC is not a substitute for regular contraception. 55.7% and 36.1% of the students were aware of levonorgestrel tablets and mifepristone respectively. 19.6% and 17.1% of the students thought IUD and vaginal douching were EC methods. 21.8% of students knew nothing about EC methods. 79.5% of students thought that having an abortion could adversely affect women's physical and mental health, and 80.6% of the students thought a history of past abortion might be a risk factor for women's future pregnancy.

**Table 2:** The knowledge of contraception and attitude towards contraception (n(%)).

		Experience of unintended pregnancy		
The knowledge and attitude	Total(n = 3,595)	Yes(n = 1,142)	No(n = 2,453)	*p* ^★^
The primary consideration for choosing contraceptive methods(missing = 96)				
Contraceptive effectiveness	1,182(33.8)	334(30.5)	848(35.3)	<0.0001
The feeling of using contraceptive methods	601(17.2)	283(25.9)	318(13.2)	
The convenience of buying or using contraceptive tools	118(3.4)	38(3.5)	80(3.3)	
The safety of contraceptive methods	1,598(45.6)	440(40.2)	1,158(48.2)	
The side effects of OCPs^a^				
Nausea/vomiting(missing = 18)	1,417(39.6)	429(38.0)	988(40.4)	0.1704
No side effects(missing = 18)	63(1.8)	44(3.9)	19(0.8)	<0.0001
If OCPs could result in the follows otions				
Affecting fertility(missing = 18)	2,225(62.2)	615(54.4)	1,610(65.8)	<0.0001
Affecting the regularity of the menstrual cycle(missing = 18)	2,451(68.5)	690(61.1)	1,761(72.0)	<0.0001
Risk of weight gain(missing = 18)	1,367(38.2)	438(38.8)	929(38.0)	0.6487
Understanding EC^b^ is not a substitute for regular contraception	2,706(75.3)	721(63.1)	1,985(80.9)	<0.0001
Understanding some recommended EC methods				
Levonorgestrel tablets(missing = 27)	1,987(55.7)	535(47.4)	1,452(59.5)	<0.0001
Mifepristone(missing = 28)	1,287(36.1)	416(36.9)	871(35.7)	0.4994
IUD^c^(missing = 28)	701(19.6)	298(26.4)	403(16.5)	<0.0001
Don’t know at all(missing = 27)	778(21.8)	208(18.4)	570(23.4)	0.0009
Understanding unrecommended EC methods				
Vaginal douching(missing = 28)	611(17.1)	244(21.6)	367(15.1)	<0.0001
The adverse effect of abortion for women's physical and mental health(missing = 17)				
Serious	2,845(79.5)	718(63.5)	2,127(86.9)	<0.0001
Slightly	447(12.5)	224(19.8)	223(9.1)	
Not at all	150(4.2)	112(9.9)	38(1.6)	
Uncertain	136(3.8)	77(6.8)	59(2.4)	
The effect of past abortion on women's future pregnancy	2,898(80.6)	830(72.7)	2,068(84.3)	<0.0001

^a^ OCPs = Oral contraceptive pills.

^b^ EC = Emergence contraception.

^c^ IUD = Intrauterine device.

^★^The comparison of the contraceptive knowledge of between the students with and without unintended pregnancy.

### The adoption frequency and methods of contraception

As shown in Table 3, the percentage of unmarried female students with sex experience who reported their frequency of contraceptive methods use as “always”, “often”, “sometimes”, “occasionally” and “never” were 28.3%, 44.3%, 11.0%, 12.7% and 3.7%, respectively. Among the female students who always adopted contraceptive methods, 285(29.0%) experienced unintended pregnancy. Among the female students who never adopt contraceptive methods, 25(19.7%) experienced unintended pregnancy. The high pregnancy rate in students who always used contraption and the low rate in those who never use contraception were not expected. Among the 25 female students with unintended pregnancy who never use contraceptive methods, 19 (76.0%) students had experienced pregnancy two or more times. Among students who always used contraception, only 40% students had experienced pregnancy two or more times. The main contraceptive method for unmarried female students who always adopted contraception was male condom (82.9%). The prevalence of unintended pregnancy was 23.2% among students who always used condoms for contraception. However, of students who chose the unrecommended contraceptive method, vaginal douching, 65.6% experienced unintended pregnancy.

**Table 3:** The types of contraceptive methods used and the frequency of adoption (n (%)).

		Experience of unintended pregnancy				
Contraceptive methods use	Total(n = 3,595)	Yes(n = 1,142)	One(n = 531)	Two and more(n = 611)	No(n = 2,453)	*p* ^★^
Frequency of contraceptive methods use	missing = 130	missing = 59	missing = 8	missing = 51	missing = 71	<0.0001
Always	982(28.3)	285(29.0)	171(60.0)	114(40.0)	697(71.0)	
Often	1,536(44.3)	446(29.0)	214(48.0)	232(52.0)	1,090(71.0)	
Sometimes	379(11,0)	145(38.3)	67(46.2)	78(53.8)	234(61.7)	
Occasionally	441(12.7)	182(41.3)	65(35.7)	117(64.3)	259(58.7)	
Never	127(3.7)	25(19.7)	6(24.0)	19(76.0)	102(80.3)	
Contraceptive methods	982(missing = 25)	285(missing = 3)	171(missing = 1)	114(missing = 2)	697(missing = 22)	
Male condom	793(82.9)	184(23.2)	113(61.4)	71(38.6)	609(76.8)	<0.0001
OCPs	472(49.3)	190(40.3)	115(60.5)	75(39.5)	282(59.7)	<0.0001
Rhythm method	255(26.7)	80(31.4)	44(55.0)	36(45.0)	175(68.6)	0.4358
Withdrawal method	340(35.5)	95(27.9)	68(71.6)	27(28.4)	245(72.1)	0.4421
Vaginal douching	93(9.7)	61(65.6)	39(63.9)	22(36.1)	32(34.4)	<0.0001
ECPs^a^	97(10.1)	44(45.4)	22(50.0)	22(50.0)	53(54.6)	0.0003

^a^ ECPs = Emergence contraceptive pills.

^★^The comparison of the contraceptive methods use between the students with and without unintended pregnancy.

### The reasons for contraception non-use

The reasons for contraception non-use were summarized in Table 4. The most common reason for not using contraception was lack of preparation (pills or devices) for sex (45.4%). There were 126 (5.2%) students who chose “didn’t know how to use contraceptive methods”, and the prevalence of pregnancy in this group was 63.5%. There was a significant difference in the reasons for contraception non-use between students with previous unintended pregnancy and those without (*p*<0.05).

**Table 4:** Reasons for not using contraceptive methods (n(%)).

		Experience of unintended pregnancy		
Reasons	Total(n = 2,613)	Yes(n = 857)	No(n = 1,756)	*p*
Believed the occasional sex could not lead to pregnancy(missing = 209)	745(31.0)	287(38.5)	458(61.5)	<0.0001
Thought contraceptive methods were too expensive to buy(missing = 205)	241(10.0)	158(65.6)	83(34.4)	<0.0001
Worried about the side effects(missing = 202)	389 (16.1)	154(39.6)	235(60.4)	0.0006
Didn’t prepare the pills or tools for the unplanned sex(missing = 201)	1,095(45.4)	286(26.1)	809(73.9)	<0.0001
Partner didn’t want (me) to use a method(missing = 201)	408(16.9)	208(51.0)	200(49.0)	<0.0001
Thought contraceptive methods were inconvenient to buy(missing = 201)	244(10.1)	98(40.2)	146(59.8)	0.0046
Thought the delight would be affected by methods(missing = 201)	687(28.5)	252(36.7)	435(63.3)	0.0025
Didn’t know how to use(missing = 201)	126(5.2)	80(63.5)	46(36.5)	<0.0001

### The latest pregnancy

As shown in Tables 5 and 6, of all unmarried female university students with unintended pregnancy, 611(53.5%) experienced twice or more pregnancies. Prior to their unintended pregnancy, 278(24.7%) female students had asked their partners to wear a male condom, 216(19.2%) took OCPs, and 408 (36.2%) did not adopt any contraceptive methods. After they unintentionally became pregnant, 55.5% of these students underwent surgical abortion 28.5% had a medical termination, 8.0% intended to carry the child while 8.0% did not have a plan at the time of survey.

**Table 5:** The types of contraceptive methods used before the latest unintended pregnancy (n = 1,142).

Types of contraceptive methods(missing = 16)	N	%
Male condom	278	24.7
OCPs	216	19.2
Rhythm method	103	9.1
Withdrawal method	90	8.0
Vaginal douching	31	2.8
No method	408	36.2

**Table 6:** The methods of terminating the latest unintended pregnancy (n = 1,142).

Methods of terminating pregnancy(missing = 246)	N	%
Surgical abortion	497	55.5
Medical abortion	255	28.5
Intending to carry a child	72	8.0
Didn’t have a plan	72	8.0

## Discussion

In China, sexual intercourse and unintended pregnancy among university students has become more common. In our study prevalence of sexual intercourse was 10.2% lower than the prevalence of 30% reported by Zhang [17]. Our sample was larger and included more regions as well as a greater range of student majors compared to the above; therefore, we believe that the prevalence of sexual intercourse reported here better represents that nationally. It is possible that our study underestimated the population prevalence due to reluctance of students to admit sexual intercourse and unintended pregnancy in a survey; because either they don’t want to tell the truth or they are “shy”. It is notable that vocational college students, as well as arts and sports students were not included in our study. One study reported a prevalence of sexual intercourse among female arts university students as high as 35% [18]. A meta-analysis might be helpful to determine the exact prevalence of sexual intercourse in unmarried female students.

The prevalence of unintended pregnancy among sexually active students in our study was 31.8%. At the first sexual intercourse, the students who were older, with higher education level and medical students had lower risk of unintended pregnancy. We may suggest that the older students and students with high education level are more mature, and may be they can protect themselves from pregnancy. Of course, the medical students have more professional contraceptive knowledge than non-medical students, and then they protect themselves well.

We found that most students (84.0%) with unintended pregnancy chose to terminate pregnancy. In contrast, one study from the United States found that most students with unintended pregnancy chose to get married; with abortion was the second most popular choice [19]. The reasons for this difference may include a difference in social and cultural background, as well as the difference in policies, regulations and laws between the USA and China. In China people are deeply influenced by traditional culture in which the premarital pregnancy is discouraged. Students in China may therefore terminate the pregnancy before it is found by others in order to protect their personal reputation. Considerations of unfinished education and unfavorable economic conditions may also play a role in their decision to terminate the pregnancy [6].

Our study suggests that even after the Chinese Ministry of Education issued a policy that university students could get married and give a birth to child provided they meet the requirements of the Marriage Law, the rate of abortion is still high among Chinese unmarried female university students. Not only unmarried female university students but unmarried women in China as a whole have a high abortion rate. Unmarried women accounted for 30% to 35% of the total number of induced abortions in China [20–22]. The cause of this might be the easy access to abortion facilities which have been built for married women of childbearing age who became pregnant outside the national family planning policy. Societal acceptance of this way of terminating unplanned pregnancy undoubtedly influences unmarried female university students’ choices; many students viewed abortion was a saviour for unintended pregnancy while largely ignoring the adverse effects of abortion.

We also found that more than half (53.5%) of the unmarried female students with unintended pregnancy had two or more pregnancies. We suggest that students with unintended pregnancy do not consider unintended pregnancy to be a serious consequence of their behavior. It is also possible that they have sexual intercourse more frequently than those who do not become pregnant. Our data agree with previous work that suggests a history of unintended pregnancy is one factor which predicts the occurrence of future unintended pregnancy [20].

Unintended pregnancy itself may cause stress to the student and interrupt their normal life. And also, abortion may cause even more harms in unmarried female university students who are not ready for pregnancy and child care. Students do not seem to realize that that abortion is a medical intervention [23], or that this can be avoided by using contraception to prevent unintended pregnancy. Our finding that 8.0% of unmarried female students with unintended pregnancy did not know how to deal with the pregnancy suggests significant proportion are not psychologically prepared to deal with this.

If used correctly contraception can prevent most unintended pregnancies; in turn reducing abortions. Our results indicate that the frequency of contraceptive methods is inversely related to the incidence of unintended pregnancy. A low prevalence of contraceptive methods use was related to high prevalence of unintended pregnancy. The majority of sexually active female students required their partners to wear condoms, with male condoms the most commonly used contraceptive methods. Similar findings were reported by Santelli JS and Zhang L [24,25]. This indicated that male condom was the most commonly used contraceptive method among Chinese university students. The prevalence of unintended pregnancy among students using condoms was lower than that among those choosing other methods, although some pregnancies still occured. If used correctly and consistently, male condoms were reported to prevent 98% of pregnancies [26]. The higher rate of unintended pregnancy in students whose male partners used condoms in our study might be due to incorrect or inconsistent use. Poor quality of condoms used may also contribute. We were surprised to find that the prevalence of unintended pregnancy was low in sexually active students who had never used contraception. One possible explanation might be that these students did not have sex so often. However, in our survey, frequency of sex was not included. This would be useful in future.

We also suggest that some students had inadequate sex education. They ignored regular contraception but adopted EC methods as remediation. It is important that education strategies highlight that EC methods are not a substitute for regular contraception, we should encourage students to use it as a backup method, but not as a regular method because of the less efficacy compared to other regular contraception [27]. The rhythm method was unreliable because of the irregularity of menstruation in some women [28]. The withdrawal method was equally unreliable due to the improper control of the extracorporeal ejaculation [29]. Vaginal douching is not reliable and refer contraception either [24]. Therefore, we suggest that the evidence-based regular contraceptive methods should be chosen and students should be educated as to the poor efficacy of the methods mentioned above.

More than 71% of student who engaged in sexual intercourse did not use contraceptive methods in every sexual contact. Two main reasons were reported for not using contraceptive. First, the sexual activity was unplanned and therefore contraception was not available. Second, students did not know that one sexual activity can lead to pregnancy. We also found that the level of contraceptive knowledge among unmarried female university students in China was poor. Similar data was reported by Zhang, et al. and Chen et al. [29,30]. A large percentage of students with unintended pregnancy did not pay enough attention to contraception and their knowledge of contraception was neither complete nor accurate. Therefore, reproduction health education is urgently needed for unmarried female students in China.

The students’ lack of knowledge makes education and consulting on sex, reproduction and contraception necessary. Parents and teachers might be best placed help to students. As early as in 1980, Canadian scholar F. Michael Barrett found that parental attitude was the main factor influencing female students’ attitude towards the sex [31]. While this suggests that discussing sex with parents may do help. In China, parents avoided discussing topics such as love, premarital sexual intercourse, contraception and abortion with their children[6]. Teachers also avoid discussing these topics with their students [6]. To make things more difficult, society as a whole is ambivalent towards providing reproductive health knowledge to the unmarried youth [32]. Efforts should therefore be made to draw the attention of parents, teachers and the society as whole to the need to improve students’ knowledge of contraception and reproduction. Students organizations and students themselves should also act proactively to promote these educational activities.

In summary, premarital sexual intercourse is relatively common among unmarried female university students in China, and unintended pregnancy and abortion among is not a rare consequence. The students lack knowledge of contraception and are not well prepared for pregnancy. Sex education in universities should be prioritized and strategies to promote consulting on sex, contraception and pregnancy should also be focused for university students.

## Supporting Information

S1 File The approval from ethics committee. (PDF)

S2 File Questionnaire of Contraception and Reproductive Health. (PDF)
