# Supplementary material for: Contraception and Unintended Pregnancy among Unmarried Female University Students: A Cross-sectional Study from China
Source: PLoS One. 2015 Jun 19;10(6):e0130212. doi: 10.1371/journal.pone.0130212 (PMC4474598; doi:10.1371/journal.pone.0130212)
Supplement: S1 File — (PDF) [file pone.0130212.s001.pdf]

华中科技大学同济医学院公共卫生学院
预防医学伦理委员会文件

伦理委员会【2006】04 号

关于"避孕节育和生殖健康适宜技术应用研究（中国大学生避孕节育和生殖健康调查）"审查批准通知

研究课题名称：避孕节育和生殖健康适宜技术应用研究（中国大学生避孕节育和生殖健康调查）

负责人：尹平教授

单位：华中科技大学同济医学院公共卫生学院

联系电话：027-83692832

合作单位：华中科技大学同济医学院计划生育研究所、重庆市人口和计划生育科学技术研究院

研究目的：本研究旨在了解中国大学生的生殖健康与避孕节育情况，为制定相关的政策提供参考意见，从而保护中国大学生的身心健康。

主要研究内容：大学生基本情况；生殖健康认知情况；避孕药具及避孕服务的利用情况以及避孕节育服务需求情况。

研究起止时间：2006 年 12 月 1 日-2009 年 12 月 31 日

调查人群数量和特征：本次调查计划于 2007 年 9 月至 2008 年 1 月，在中国的北京、长春、成都、重庆、南京、南宁及武汉 7 个城市，利用分层整群抽样的方法，对 49 所全日制高校的大学生以无记名方式进行问卷调查，计划调查 78400 名大学生。

道德伦理委员会意见

该课题研究目的、计划研究方案和实施过程均符合道德伦理委员会的要求，同意该课题进行。并请严格按照申请书内容运作。

同济医学院公共卫生学院道德伦理委员会主任：

2006 年 12 月 25 日

Tongji Medical College (TMC)

INSTITUTIONAL REVIEW BOARD (IRB) APPROVAL

<table>
<tr><td>Protocol Title</td><td>A Cross-sectional Study of Contraception and Reproduction Health among Chinese University Students</td></tr>
<tr><td>Name of primary investigator in TMC</td><td>Ping YIN</td></tr>
<tr><td>Division
Telephone</td><td>Department of Epidemiology and Biostatistics, School of Public Health, Tongji Medical College, Huazhong University of Science and Technology +86-27-83692832; 13098818499</td></tr>
<tr><td>Outside investigators involved in study</td><td>Institute of Family Planning, Tongji Medical College, Huazhong University of Science and Technology; Institute for Population and Family Planning of Chongqing City, China</td></tr>
<tr><td colspan="2">Summary of research:
Background: The premarital sex behavior and unintended pregnancy of Chinese university students increase day by day. This study was to improve the cognitive level of contraception and reproduction health, and also the awareness of unhealthy sex of Chinese university students in order to protect their physical and mental health.

Methods: A cross-sectional study would be conducted from September 2007 to January 2008, in which the college students were investigated in 49 universities from 7 cities in different regions of China. The content of this study included the basic demographic characteristics, cognitive level of contraception and reproduction health, contraceptive methods use and the needs of contraceptive service.</td></tr>
<tr><td>Proposed Project Dates</td><td>12/01/2006-12/31/2009</td></tr>
<tr><td>Study population</td><td>The population of this study is the university students from different regions of China.</td></tr>
<tr><td>The views of IRB</td><td>Approval.
Please conduct strictly according to your protocol.</td></tr>
<tr><td>Director
TMC IRB
Date</td><td>Tangchun Wu
Prof. Tangchun Wu, Dec.25, 2006</td></tr>
</table>
