# Supplementary material for: Contraception and Unintended Pregnancy among Unmarried Female University Students: A Cross-sectional Study from China
Source: PLoS One. 2015 Jun 19;10(6):e0130212. doi: 10.1371/journal.pone.0130212 (PMC4474598; doi:10.1371/journal.pone.0130212)
Supplement: S2 File — (PDF) [file pone.0130212.s002.pdf]

Knowledge,Attitude and Practice Questionnaire about Contraception and Reproductive Health of University

Part 1 Basic information

**A1**	Sex：1-Male 2-Female	□
**A2**	Birth(**year/month/day**)	□□/□□/ □□
**A3**	Race： 1-Han<br>2-Minority (please specify)	□<br>2____________
	What is your education certificate?<br>1- freshman year 2-sophomore 3-junior 4- senior<br>5- The fifth grade at university 6- first-year of graduate<br>7- second-year of graduate 8-third-year of graduate<br>9- first-year of doctor 10- second-year of doctor<br>11-third-year of doctor	□
**A4**	Your major is：<br>1-History class<br>2-Science and Technology<br>3-Medicine<br>4-Agronomy<br>5-Sports<br>6-Arts	□
**A5**	Do you have a lover now or ever? 1-Yes 2-No	□
**A6**	Marital status：1-Unmarried 2-Married 3-Divorced 4- Widowed	□

**A7**	How much is your monthly expenses(Yuan):	□□□<br>□

Part 2 Knowledge and Attitude

**B1**	What are the basic conditions for human pregnancy？（multiple choice）<br>1-Sperm 2-Ovun<br>3- Genital tract 4-Uterus	□1□2<br>□3□4
**B2**	Which following is true about menstruation:<br>1-After ovulation will have menstruation<br>2- The temperature is rising in menstrual period<br>3-Ovulation happens on the 14th day before next menstruation<br>4- Ovulation happens on the 14th day behind menstruation	□
**B3**	which following is male genitalia？（multiple choice） 1-Penis<br>2-Prostate 3-Bladder 4-Ovary 5-Epididymis 6-Paranephros	□1□2□3□4<br>□5□6
**B4**	What do you think it is necessary to know the contraceptive knowledge<br>1- Necessary 2- Unnecessary	□
**B5**	Who should be response for contraception?<br>1- Man 2-Woman<br>3-Both of them have responsibility<br>4- Both of them without responsibility	□
**B6**	The priority consideration of choosing contraceptive methods：<br>1-Contraceptive effectiveness<br>2-The feeling of using contraceptive methods<br>3-The convenience of buying or using contraceptive tools	□

	4- The safety of contraceptive methods	
**B7**	The side effects of Oral contraceptive pills？（multiple choice）<br>1-Affecting fertility<br>2-Affecting the regularity of the menstrual cycle<br>3- Risk of weight gain<br>4-Nausea/vomit<br>5- No side effects	□1□2□3□4<br>□5□6□7□8
**B8**	Which methods can used for emergency contraception？（multiple choice）<br>1- levonorgestrel tablets 2- mifepristone<br>3- Intrauterine device 4- Vaginal douching<br>5- Don't know at all	□1□2□3□4<br>□5
**B9**	Whether emergency contraception can substitute for regular contraception？<br>1-Can 2-Can not 3-Don't know	□
**B10**	Do you think contraceptive method can completely contraception？ 1-Can<br>2-Can not 3- Don't know	□
**B11**	Which contraceptive methods do you think is suitable for college students？(multiple choice）<br>1- Rhythm method 2- Intrauterine device<br>3- Oral contraceptive pills 4- Norplant<br>5-EC method 6- Withdrawal<br>7-Male condom 8-Female condom<br>9- Contraceptive vaginal ring 10-Don't know at all	□1□2□3□4<br>□5□6□7□8<br>□9□10
**B12**	Which stage of the menstrual cycle most likely to conceive？<br>1-Menstrual period<br>2- A few days before or after menstruation<br>3-About 14 days before menstruation<br>4- Don't know at all	□
**B13**	Do you think there may be an impact on women's physical and mental health after abortion?<br>1- Not at all	□

	2- Slightly<br>3- Serious<br>4- Uncertain	
**B14**	Do you think there may be an impact on women pregnancy after abortion?<br>1-Yes 2-No	□
**B15**	Do you think university students need to learn sexual knowledge？ 1-Need 2-Do not need	□
**B16**	Do you agree with "sex education will lead to more sexual behavior"? 1-Agree 2-Disagree	□
**B17**	How do you think about "chastity"：<br>1-Important for female but not important for male<br>2-Important for male but not important for female<br>3-It is important for both male and female<br>4-It is not important for both male and female	□
**B18**	Do you agree with the popular concept of “sexual revolution”：<br>1-Agree 2-Disagree 3- Indifferent	□
**B19**	What is your attitude toward premarital sexual behavior：<br>1- Don't agree with premarital sex<br>2- If I have a boyfriend/girlfriend, I can accept<br>3- If I am ready to get married with he/she, I can accept<br>4- No matter I have emotion for he/she, I can accept	□
**B20**	What is your attitude toward unmarried cohabitation：<br>1-Agree 2- Can understand 3-Disagree	□
**B21**	Have you ever had sexual impulses？<br>1-Always 2- Occasionally 3-None	□
**B22**	Have you ever had masturbation？<br>1-Always 2- Occasionally 3-None	□
**B23**	Do you think masturbation is normal?<br>1-Normal 2-Unnormal	□
**B24**	Which following are sexually transmitted disease？（multiple choice）<br>1-Syphilis 2-AIDS 3-Non gonococcal urethritis<br>4-Gonorrhea 5- Condyloma acuminatum 6- Genital herpes<br>7- Hepatitis B 8- None of the above 9-Don’t know at all	□1□2□3<br>□4□5□6<br>□7□8□9

**B25**	Which way can transmit AIDS?(multiple choice) 1-Blood transfusions<br>2- Sharing contaminated needles<br>3-Sharing food<br>4-Shaking hands or sharing personal objects<br>5-Unprotected sex<br>6-Kissing	□1□2□3<br>□4□5□6

Part 3 Practice

**C1**	Have you ever had sex behavior（sex intercourse）？<br>1-Yes, in the recent half year<br>2-Yes， In half a year ago<br>3-No	□
**C2**	How old do you have your first sexual behavior?：□□	□□
**C3**	Did you use contraception during your first sexual behavior?<br>1-Yes 2-No (skip to C5)	□
**C4**	Which contraceptive method you for your first sexual behavior?<br>1-Condom 2-Oral contraception pills 3-Rhythm method<br>4-Withdraw 5-Intrauterine device 6-Noplant	□
**C5**	Have you or your sex partner ever had unintended pregnancy？<br>1-Yes, in the recent half year<br>2-Yes， In half a year ago<br>3-no (skip to C9)	□
**C6**	How many times do you or your sex partner have unintended pregnancy<br>:<br>1-Once 2-Twice 3-≥Three times	□
**C7**	Which method of contraception in your latest unintended pregnancy<br>1-Condom 2- Oral contraception pills 3- Rhythm method<br>4- Withdraw 5-Vaginal douching 6-Intrauterine device<br>7- Norplant 8- No method	□

**C8**	How do you deal with the latest unintended pregnancy：<br>1-Surgical abortion<br>2-Medical abortion<br>3-Preparing to give birth to child<br>4- Didn't know how to deal with it	□
**C9**	Frequency of contraceptive use?<br>1-Always 2- Often 3- Sometimes<br>4- Occasionally 5-Never	□
**C10**	Which contraception method you ever used？（multiple choice）<br>1-Condom 2- Oral contraception pills 3- Rhythm method<br>4- Withdraw 5- Vaginal douching 6-Intrauterine device<br>7- Norplant	□1□2□3<br>□4□5□6<br>□7□8□9
**C11**	Why don't you use contraception（multiple choice）<br>1-Thought the occasional sex could not lead to pregnancy<br>2-Thought contraceptive methods were too expensive to buy<br>3-Worried about the side effects<br>4-Didn't prepare the pills or tools for the unplanned sex<br>5-Partner didn't want (me) to use a method<br>6- Thought contraceptive methods were inconvenient to buy<br>7-Thought the delight would be affected by methods<br>8- Didn't know how to use	□1□2□3<br>□4□5□6<br>□7□8□9
**C12**	Which way do you obtain the sex and contraception knowledge（multiple choice）<br>1- Popular science readings<br>2- Newspaper and periodicals<br>3- Network<br>4- Radio and TV<br>5- Classmates and friends<br>6- Course education<br>7- Family<br>8- The family planning professionals<br>9- Lectures<br>10- Medical staff<br>11- Exhibition<br>12- Informal publications	□1□2□3□4<br>□5□6□7□8<br>□9□10□11□12
